# Thoughts about sex and gender differences from the next generation of autism scientists

**DOI:** 10.1186/s13229-015-0046-8

**Published:** 2015-09-17

**Authors:** Lauren Singer

**Affiliations:** Scarsdale High School, 1057 White Plains Road, Scarsdale 10583 NY, USA

## Abstract

According to the CDC, males are four times more likely to be diagnosed with autism than females. New studies have shown that girls need a higher burden of genetic mutation to be diagnosed with autism than males. These findings are leading researchers to a new avenue of investigation called the female protective effect. This theory holds that even when females carry mutations in autism-linked genes, the effect of the mutations is prevented when the level of genetic disruption is low. Understanding the biology behind this protective effect and studying females independently from males could lead to major advancements in the prevention and treatment of ASD in both males and females.

When I began my high school’s science research program last year, I knew that I wanted to study autism. My older sister Jodie was diagnosed with autism at age two, and for 15 years, I have been called her “unaffected sibling.” I have gone to walks, raised money via lemonade stands, volunteered in respite programs for families with kids with autism, and participated in autism research studies at the Yale Child Study Center, but now I have begun what I hope will be my own career as an autism research scientist.

I have experienced firsthand the anguish autism inflicts on the entire family. Many kids with the condition, including my sister, are non or minimally verbal and have aggressive and self-injurious behaviors. The Centers for Disease Control and Prevention (CDC) report that 40 % of those with autism are also intellectually disabled [1]. Symptoms range from moderate to very severe, and people with profound autism, like my sister, often need around-the-clock care to keep them safe. Our house was always chaotic with therapists coming and going. Nighttime was stressful and challenging, as Jodie never seemed to need sleep. She kicked the bedroom wall we shared and made all kinds of noises that kept everyone in my family awake. Several times she hit or bit me unprovoked. My parents worked out a system so that one of them was always with her. Last year, at age 17, Jodie’s behavior became so aggressive that she could no longer live safely at home, and so she moved into residential placement. Living with Jodie, and now having to live without her, has made me desperate for answers.

In the inaugural thematic series of Molecular Autism published earlier this year, editors Meng-Chuan Lai, Simon Baron-Cohen, and Joseph Buxbaum acknowledge the need to “bring sex and gender to the center stage of autism research” [2]. According to the CDC, males show higher rates of autism than females [1], although a portion of this difference may be due to diagnosis bias [2]. As I began my high school science research classes, I knew I wanted to focus on these gender differences because, according to Halladay and colleagues, “better understanding of sex differences could lead to major advancements in the prevention and treatment of ASD in both males and females” [3]. For years, researchers looking at this gender disparity assumed that autism must be hormone or X-chromosome linked. One study, published by Baron-Cohen and colleagues in 2014, showed that children who later developed autism were exposed to elevated levels of steroid hormones, such as testosterone, progesterone, and cortisol during the prenatal period, based on amniotic fluid analysis [4]. The authors posit that because some of these hormones are produced in much higher quantities in males than in females, this may help explain why autism is more common in males. Meanwhile, geneticists studying this disorder found that autistic children had a consistent pattern of de novo genetic mutations that might be causing their condition [5–10]. As scientists discovered these genes, they found something surprising: girls with autism showed a higher burden of mutation than males with autism [5, 7, 11, 12]. Additionally, some girls with genetic mutations did not show clinical features of autism, indicating that they had greater resistance to autism from genetic causes than boys did [12].

These findings are leading researchers to a new avenue of investigation called the “Female Protective Effect (FPE).” The theory holds that this protective factor is strong enough to shield girls who have a minimal to moderate level of genetic disruption, but for girls with a large genetic burden for autism, the protective factor cannot compensate [11]. This explains why girls usually have more severe symptoms [6, 10, 11]. For example, Frazier et al. report that among individuals with autism who have lower IQ, girls experience greater social communication impairments than boys. Girls in this group also have lower IQs on average than boys [13]. If a female protective factor is discovered and isolated, it could be harnessed to protect all children from the debilitating features of autism. I hope that the research will continue to advance so that scientists can create a medication mimicking the properties of the protective factor that will alleviate everyone’s autism symptoms.

Lai, Baron-Cohen, and Buxbaum also explain that females are underrepresented in research, and therefore, in the scientific and clinical literature [2]. In the future, in addition to including more females, it will be important to analyze autistic females’ responses to treatment separately from those of autistic males. Due to their underlying genetic, biological, and brain-based differences [14], girls with autism may respond differently to treatment than boys with autism. If males and females are analyzed together, different responses from females may be masked by the responses of the larger male group. I am hoping to focus my research on looking at treatment response differences among males and females with autism.

I also believe that scientists can enhance their careers by leaving the lab once in a while to interact with real people with autism. For example, seeing my sister struggle to remember peoples’ names and faces enabled me to understand the real impact that a social memory-enhancing drug could have on a person’s life. Having volunteered in a respite program for 4 years now, I have seen what the most debilitating aspects of autism are in terms of social functioning, and it has made me aware of the types of medications that might be most beneficial. It is much easier to see this in people than in rats. Seeing people with autism challenged by daily living skills can make research designed to help them feel much more rewarding. Although animal models can mimic the clinical symptoms of autism, everyone should spend time with actual people; people are more than the sum of their gene sequences.

Living with Jodie has taught me to appreciate hard work and incremental improvement. When Jodie was in applied behavior analysis (ABA) therapy for 40 hours per week, I did not understand how difficult it must have been for her. Therapists came and went throughout the day, but Jodie had to stay and work with each one. She struggled to learn concepts such as colors, shapes, and numbers that came easily to me and to others. And when she mastered a new skill, my family celebrated as much as if she had been accepted to college. There are good scientific studies supporting the value of ABA, but it is important for scientists to continue to develop new therapies so that kids can gain as many new skills as possible.

The word “autism” entered my vernacular long before I was in kindergarten, when my mom first started explaining why my sister never answered when I spoke to her, never let me hug her, and, in fact, seemed unable to even look at me. When I was 3 years old, I perceived autism as a cold that Jodie had that would eventually go away. As I began to develop a better grasp of autism, I felt a need to explain Jodie’s bizarre behavior to friends and even strangers who stared at her. Now, I finally have the opportunity to do more than simply increase awareness. This summer, I began my work in autism research as an intern at the Seaver Autism Center at the Icahn School of Medicine at Mount Sinai Hospital. I hope eventually to be part of the scientific research team that discovers how the female protective factor may be the foundation of a new treatment. Halladay and colleagues describe that “under the FPE model, a higher rate of ASD recurrence is expected in the siblings of affected females than the siblings of affected males, a phenomenon often called the “Carter effect” [3]. This means that as the sibling of a female with ASD, I was at even greater risk for autism. I sometimes wonder why Jodie is so severely affected by autism, while I am not, given that as sisters, we may share the same risk-increasing genetic variants. I may be able to be tested to see if I carry any autism mutations or the protective factor, but whether or not I turn out to be a “carrier female,” I am clearly not an “unaffected sibling.” My experiences with Jodie and my hopes for her future have inspired me to want to be part of the cutting edge of discovery that will make a difference in the lives of thousands of people.

## Abbreviations

ABA: applied behavior analysis
ASD: autism spectrum disorder
CDC: The Centers for Disease Control and Prevention
FPE: female protective effect

## References

[1] Developmental Disabilities Monitoring Network Surveillance Year Principal, I, C (2014). Centers for Disease, and Prevention, Prevalence of autism spectrum disorder among children aged 8 years - autism and developmental disabilities monitoring network, 11 sites, United States, 2010. MMWR Surveill Summ.

[2] Lai MC, Baron-Cohen S, Buxbaum JD (2015). Understanding autism in the light of sex/gender. Mol Autism.

[3] Halladay AK, Bishop S, Constantino J, Daniels A, Koenig K, Palmer K (2015). Sex and gender differences in autism spectrum disorder: summarizing evidence gaps and identifying emerging areas of priority. Mol Autism.

[4] Baron-Cohen S, Auyeung B, Norgaard-Pedersen B, Hougarrd DM, Abdallah MW, Melgaard L (2015). Elevated fetal steroidogenic activity in autism. Mol Psychiatry.

[5] De Rubeis S, He X, Goldberg AP, Poultney CS, Samocha K, Cicek AE (2014). Synaptic, transcriptional and chromatin genes disrupted in autism. Nature.

[6] Iossifov I, O'Roak BJ, Sanders SJ, Ronemus M, Krumm N, Levy D (2014). The contribution of de novo coding mutations to autism spectrum disorder. Nature.

[7] Iossifov I, Ronemus M, Levy D, Wang Z, Hakker I, Rosenbaum J (2012). De novo gene disruptions in children on the autistic spectrum. Neuron.

[8] Neale BM, Kou Y, Liu L, Ma'ayan A, Samocha K, Sabo A (2012). Patterns and rates of exonic de novo mutations in autism spectrum disorders. Nature.

[9] O’Roak BJ, Vives L, Girirajan S, Karakoc E, Krumm N, Coe B (2012). Sporadic autism exomes reveal a highly interconnected protein network of de novo mutations. Nature.

[10] Sanders SJ, Murtha M, Gupta A, Murdoch J, Raubeson M, Willsey AJ (2012). De novo mutations revealed by whole-exome sequencing are strongly associated with autism. Nature.

[11] Jacquemont S, Coe BP, Hersch M, Duyzend MH, Krumm N, Bergmann S (2014). A higher mutational burden in females supports a “female protective model” in neurodevelopmental disorders. Am J Hum Genet.

[12] Levy D, Ronemus M, Yamrom B, Lee YH, Leotta A, Kendall J (2011). Rare de novo and transmitted copy-number variation in autistic spectrum disorders. Neuron.

[13] Frazier TW, Georgiades S, Bishop SL, Hardan AY (2014). Behavioral and cognitive characteristics of females and males with autism in the Simons Simplex Collection. J Am Acad Child Adolesc Psychiatry.

[14] Nordahl CW, Iosif A, Young GS, Perry LM, Dougherty R, Lee A (2015). Sex differences in the corpus callosum in preschool-aged children with autism spectrum disorder. Mol Autism.

